# Intensification of Pseudocapacitance by Nanopore Engineering on Waste-Bamboo-Derived Carbon as a Positive Electrode for Lithium-Ion Batteries

**DOI:** 10.3390/ma12172733

**Published:** 2019-08-26

**Authors:** Jong Chan Hyun, Jin Hwan Kwak, Min Eui Lee, Jaewon Choi, Jinsoo Kim, Seung-Soo Kim, Young Soo Yun

**Affiliations:** 1 Department of Chemical Engineering, Kangwon National University, Samcheok 25913, Korea; 2 Institute of Advanced Composite Materials, Korea Institute of Science and Technology (KIST), Jeonbuk 55324, Korea; 3 Department of Chemical Engineering, Kyung Hee University, Yongin 17104, Korea

**Keywords:** nanopore, pseudocapacitor, cathode, porous carbon, lithium-ion, batteries

## Abstract

Nanoporous carbon, including redox-active functional groups, can be a promising active electrode material (AEM) as a positive electrode for lithium-ion batteries owing to its high electrochemical performance originating from the host-free surface-driven charge storage process. This study examined the effects of the nanopore size on the pseudocapacitance of the nanoporous carbon materials using nanopore-engineered carbon-based AEMs (NE-C-AEMs). The pseudocapacitance of NE-C-AEMs was intensified, when the pore diameter was ≥2 nm in a voltage range of 1.0~4.8 V vs Li^+^/Li under the conventional carbonate-based electrolyte system, showing a high specific capacity of ~485 mA·h·g^−1^. In addition, the NE-C-AEMs exhibited high rate capabilities at current ranges from 0.2 to 4.0 A·g^−1^ as well as stable cycling behavior for more than 300 cycles. The high electrochemical performance of NE-C-AEMs was demonstrated by full-cell tests with a graphite nanosheet anode, where a high specific energy and power of ~345 Wh·kg^−1^ and ~6100 W·Kg^−1^, respectively, were achieved.

## 1. Introduction

Lithium-ion batteries (LIBs) are widely used power sources ranging from compact electronic devices to the grid system, and their applications are increasing rapidly with advances in modern technology, such as internet of things, wearable electronic devices, drones, and electric vehicles [1,2,3,4,5]. The rapidly growing markets require higher energy/power densities and longer cycle lives, but the current LIB system has limited electrochemical performance [6,7]. In particular, intercalation-based host cathode materials provide restricted active sites in rigid crystal lattices composed of heavier elements, leading to insufficient specific capacities of <~200 mA·h·g^−1^ [8]. Although conversion-type cathode materials, such as S, CuCl_2_, BiF_3_, and FeF_3_, show remarkably improved specific capacities, they suffer from critical obstacles, such as large voltage hysteresis, low round-trip efficiency, and poor cycling stability [8]. Consequently, the development of high-performance cathode materials is one of the key issues in producing more advanced LIB systems.

Carbon-based active electrode materials (C-AEMs) composed of multiple polyhexagonal carbon (PHC) building blocks have an idiosyncratic nature arising from the sp^2^-conjugated bonding structure and aromaticity [9,10,11,12]. They can readily donate or accept a delocalized electron with charge compensation caused by counter-ion adsorption on the surface of PHCs [13,14,15]. In addition, their high specific surface areas and high electrical conductivities enable significantly fast and stable charge delivery [16,17,18,19]. A more interesting aspect of the C-AEMs is the surface-driven faradic behaviors in the cathodic voltage region of >2 V [20,21,22,23,24,25,26]. A previous study reported that the pseudocapacitive charge storage of C-AEMs is induced partly by the synergistic redox behaviors of the neighboring two heteroatoms, such as oxygen, nitrogen, and sulfur [20]. The relationship between the heteroatom contents and specific capacity was also demonstrated using graphene nanosheets with engineered oxygen functional groups. High specific capacities of 250 mA·h·g^−1^ were achieved in the positive voltage range, when doped with oxygen heteroatoms up to an oxygen-to-carbon atomic ratio of ~0.47 [21]. These results suggest that the specific capacities can be increased by extending the active surface area, including heteroatoms. On the other hand, the relationship cannot be understood by simple linear behavior because all the active surfaces in the highly complex microstructure of C-AEMs could not be exposed to the charge carriers in the electrolyte. This means that the redox-active heteroatoms can work under specific conditions with an effective active surface area (EASA) that is accessible to charge carriers.

In this study, nanopore-engineered C-AEMs (NE-C-AEMs) were prepared from a waste bamboo (WB) by carbonization and followed by chemical activation to clarify the effects of nanopores on pseudocapacitive charge storage. The pore structure and surface properties of the NE-C-AEMs were controlled using an activation agent, resulting in a series of four well-defined samples. Unlike the general belief that the specific capacity can be enhanced by increasing the specific surface area/heteroatom contents, in this study, a micropore-dominant sample with a high specific surface area showed poor specific capacity, despite its high heteroatom content. In contrast, a slight increase in micropore size up to ~2 nm resulted in considerable enhancement of the specific capacity by ~485 mA·h·g^−1^ in a positive voltage section for LIBs. This indicates a strong interrelationship between the nanopore size and electrochemical performance on C-AEMs. Moreover, NE-C-AEMs demonstrated their competitive electrochemical performance in both half-cell and full-cell tests. 

## 2. Experimental

### 2.1. Preparation of NE-C-AEMs

WBs were chopped into several millimeter-sized fragments and treated thermally from room temperature to 600 °C at a heating rate of 5 min·min^−1^ with a holding time of 2 h at the final temperature under an Ar atmosphere. The carbonized WBs were mixed with potassium hydroxide in a mortar at mass ratios of 200, 400, 600, and 800 wt.% KOH. The mixtures were heated from room temperature to 800 °C at a heating rate of 5 °C·min^−1^, which was followed by holding for 2 h at 800 °C under an Ar flow of 300 mL·min^−1^. After cooling naturally to room temperature, the samples were washed several times with excess ethanol and distilled water. The products, NE-C-AEMs, were then dried in a vacuum oven at 30 °C.

### 2.2. Characterization

Sample morphologies were observed by FE-SEM (S-4300SE, Hitachi, Tokyo, Japan) and FE-TEM (JEM2100F, JEOL, Tokyo, Japan). XRD (DMAX 2500, Rigaku, Tokyo, Japan) was conducted using a Cu Kα radiation generator (λ = 0.154 nm) at 40 kV and 100 mA with a 2θ range of 5°–60°. The Raman spectra were recorded using a continuous-wave linearly polarized laser (514.5 nm, 2.41 eV, 16 mW). The laser beam was focused using a 100× objective lens, resulting in a 1 μm spot diameter. The chemical composition and depth profile were examined by XPS (PHI 5700 ESCA, Chanhassen, MN, USA) with monochromatic Al Kα radiation. The electrical conductivity of the MP-NWBs was tested using an electrical conductivity meter (Loresta GP, Mitsubishi Chemical, Tokyo, Japan). The pore structure of the samples was analyzed using N_2_ adsorption and desorption isotherms obtained using a surface area and porosimetry analyzer (ASAP 2020, Micromeritics, Norcross, GA, USA) at −196 °C.

### 2.3. Electrochemical Characterization

The electrochemical properties of the NE-C-AEM cathodes, GNS anodes, and GNS//NE-C-AEM full cells were investigated using a Wonatech automatic battery cycler and CR2032-type coin cells. For the half-cell experiments, the coin cells were assembled in a glovebox filled with argon using the AEMs as the working electrodes and metallic Li foil as the reference and counter electrodes. LiPF_6_ (1 M; Sigma-Aldrich, 99.98%) was dissolved in a mixture solution of ethylene carbonate (EC) and dimethyl carbonate (DMC) (1:1 v/v) and used as the electrolyte. A glass microfiber filter (GF/F, Whatman) was used as a separator. The working electrodes were prepared by mixing the active material (70 wt.%) with conductive carbon (20 wt.%) and polyvinylidene fluoride (10 wt.%) in N-methyl-2-pyrrolidone. The resulting slurries were applied uniformly to the Cu/Al foils. The electrodes were dried at 120 °C for 2 h and roll pressed. The active materials of anode and cathode were loaded at densities of ~1 mg·cm^−2^ (about 2 mg for both anode and cathode), and the full cell was assembled using ~2 mg of anode and cathode as the same loading contents.

## 3. Results and Discussion

**Figure 1:**
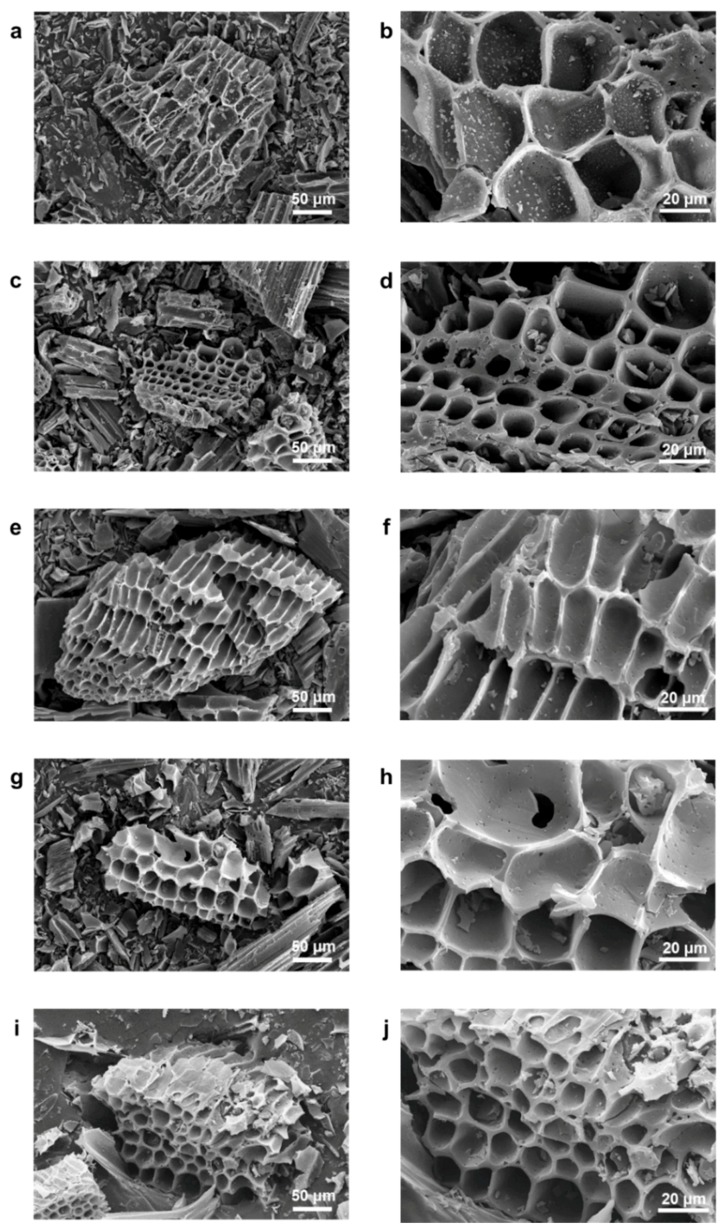
FE-SEM images of (**a**,**b**) WB, (**c**,**d**) NE2-C-AEMs, (**e**,**f**) NE4-C-AEMs, (**g**,**h**) NE6-C-AEMs, and (**i**,**j**) NE8-C-AEMs characterized in different magnifications.

**Figure 2:**
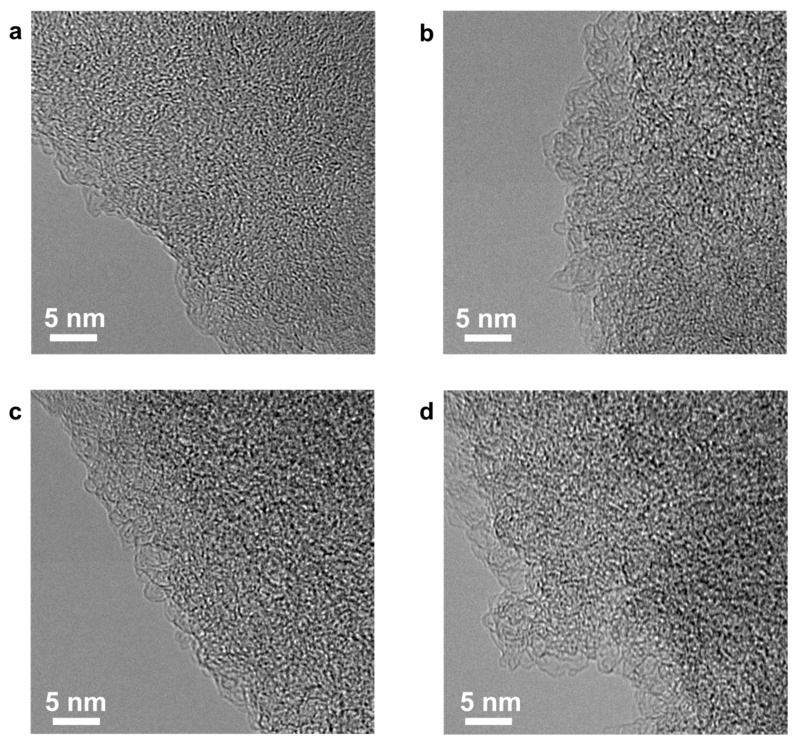
High-resolution FE-TEM images of (**a**) NE2-C-AEMs, (**b**) NE4-C-AEMs, (**c**) NE6-C-AEMs, and (**d**) NE8-C-AEMs.

**Figure 3:**
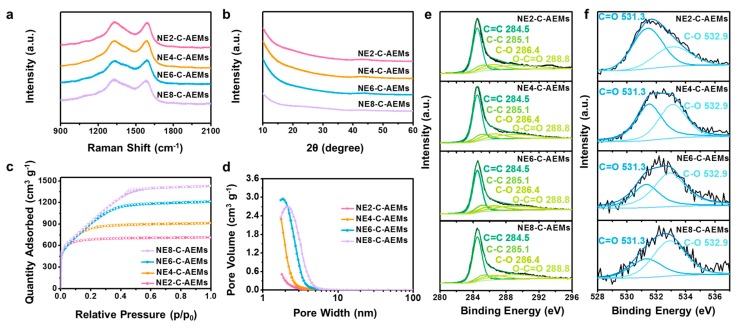
Material properties of the NE-C-AEMs. (**a**) Raman spectra, (**b**) XRD patterns, (**c**) Nitrogen adsorption and desorption isotherm curves, (**d**) Pore size distribution data, and XPS (**e**) C 1s and (**f**) O 1s spectra.

In a chemical activation process, four different samples were prepared by controlling the contents of the activation agent from 200 to 800 wt.% (vs contents of the precursor material). The resulting products are called NE2-C-AEMs, NE4-C-AEMs, NE6-C-AEMs, and NE8-C-AEMs according to the weight ratio of the activation agent to the precursor. The morphologies of the NE-C-AEMs were similar to their precursor materials because the cellulose-based precursors were a char-type material that does not undergo a melting phase during the thermal transition process (Figure 1). In addition, they all showed a highly disordered carbon structure without long-range ordering, as shown in the high-resolution field emission transmission electron microscopy (FE-TEM) images (Figure 2). The disordered structures were examined further by Raman spectroscopy and X-ray diffraction (XRD) (Figure 3a,b). In the Raman spectra, the four different samples exhibited two distinct connected peaks corresponding to *D* (A_1g_ breathing mode) and *G* (E_2g_ vibration mode) bands at ~1330 and ~1600 cm^−1^, respectively (Figure 3a). The broad and fused peaks indicated the presence of highly defective PHC structures [27]. In contrast, their XRD patterns showed no distinctive peak, indicating poor three-dimensional graphitic ordering (Figure 3b). Basically, carbon-based materials prepared from a polymer-based precursor material using a relatively low temperature pyrolysis process (<1000 °C) have a highly amorphous structure composed of defective polycrystalline PHC building blocks [28]. The sp^2^-structured PHC components were damaged further by metallic potassium-mediated chemical activation, leading to the formation of a large number of sp^3^ carbon rings, such as pentagons, hexagons, heptagons, and so forth. The topological defects formed in the PHC structures highly distorted the two-dimension-like PHCs, causing numerous (sub-)nano-meter-sized pores between the not well-stacked PHCs [29,30]. Although it is difficult to exteriorize the defective carbon structures by FE-TEM, Raman spectroscopy, and XRD, it is clear that the pore structures of C-AEMs are altered significantly by the degree of activation.

As shown in Table 1, the specific Brunauer–Emmett–Teller (BET) surface areas of NE-C-AEMs were increased gradually with increasing content of the activation agent, with NE8-C-AEMs showing the maximum surface area of ~3650 m^2^·g^−1^. In addition, the total pore volume and average pore radius increased gradually from 1.09 cm^3^·g^−1^ and 3.65 Å to 2.20 cm^3^·g^−1^ and 6.40 Å, respectively, whereas the average PHC size decreased gradually with increasing activation agent content: 23.4, 20.6, 17.7, and 16.4 Å for NE2-C-AEMs, NE4-C-AEMs, NE6-C-AEMs, and NE8-C-AEMs, respectively. Although it is difficult to believe that the particle size obtained is the absolute sum of the PHC building blocks, the results suggest a relationship between the particle size and activation agent ratio. In addition, a smaller particle size indicates the development of more carbon edge sites. The pore structure of NE-C-AEMs was analyzed further from nitrogen adsorption and desorption isotherm curves (Figure 3c). The isotherm curves showed a similar monolayer adsorption volume corresponding to approximately 500 cm^3^·g^−1^ in a relative pressure area of <0.02. This suggests that all the NE-C-AEMs have a similar open specific surface area for monolayer adsorption. As shown in Table 1, the specific micropore surface area and specific volume were 2143~2317 m^2^·g^−1^ and 0.919~0.987 cm^3^·g^−1^, respectively. In contrast, a large difference was observed in the low-medium relative pressure (LMP) section between 0.02 and 0.5. With increasing activation agent ratio, the specific adsorption volume was increased gradually in the LMP section. The sloping increase originated from the multilayer adsorption of nitrogen molecules. On the other hand, the isotherm curves indicated no hysteresis between the adsorption and desorption curves. This is in contrast to the pore-filling behavior observed in the mesopores accompanying a hysteresis. In other words, the adsorption behavior represented in the LMP section was distinct from that observed in typical micropores or mesopores. The pore size distribution data (pore diameter vs pore volume) showed that most of the pores in NE-C-AEMs were below ~4 nm in size (Figure 3d). The pore size increased gradually with increasing activation agent ratio, indicating that the nanopores can be tunable on a few-nanometer scale by controlling the activation process. As a result, it was confirmed that the large specific adsorption volume in the LMP section originated from ~2 nm pores. Furthermore, the mesopore surface area of NE-C-AEMs changed significantly according to the activation agents (245.5, 593.1, 1252.9, and 1334.0 m^2^·g^−1^ for NE2-C-AEMs, NE4-C-AEMs, NE6-C-AEMs, and NE8-C-AEMs, respectively). Table 1 lists the specific textural properties as confirmation.

The surface properties of the NE-C-AEMs were investigated by X-ray photoelectron spectroscopy (XPS), as shown in Figure 3e,f. The deconvoluted XPS C 1*s* spectra revealed the four-series samples to have similar surface functional groups composed of main sp^2^ C=C bonding and minor sp^3^ C–C, C–O, and O–C=O bonding (Figure 3e). The relative C–O to C=O bonding intensity ratio was increased gradually with increasing amount of activation agents (Figure 3f). The C/O ratios increased gradually with increasing activation agent contents: 5.9, 10.6, 15.1, and 28.1 for NE2-C-AEMs, NE4-C-AEMs, NE6-C-AEMs, and NE8-C-AEMs, respectively. This indicates that oxygen functional groups are removed gradually under harsher activation conditions. Elemental analysis also revealed a similar tendency on the weight ratio between carbon and oxygen (Table 2). The carbon-to-oxygen weight ratio increased from 5.1 to 22.7 with increasing activation agent content. As the activation conditions become increasingly severe, the oxygen functional groups in the PHC building blocks could be emitted as flue gas, such as CO and CO_2_, resulting in a decrease in oxygen contents. Table 2 lists the detailed heteroatom contents and their ratio.

**Figure 4:**
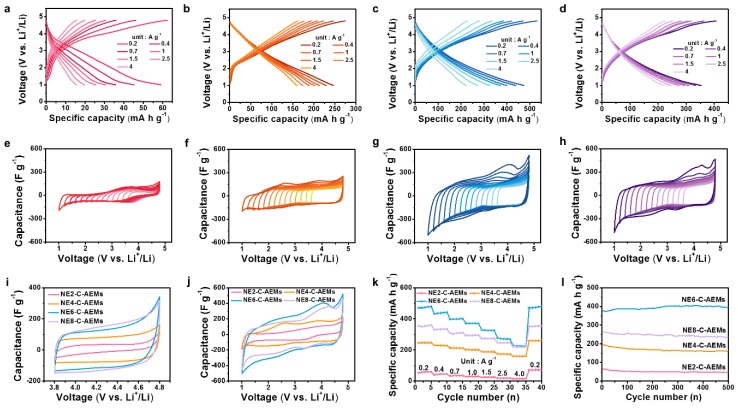
Electrochemical properties of the NE-C-AEMs in an electrolyte of 1 M LiPF6 dissolved in an EC/DMC mixture solution (1:1 v/v) as a positive electrode for LIBs. Galvanostatic charge/discharge profiles of (**a**) NE2-C-AEMs, (**b**) NE4-C-AEMs, (**c**) NE6-C-AEMs, and (**d**) NE8-C-AEMs. CV curves of (**e**) NE2-C-AEMs, (**f**) NE4-C-AEMs, (**g**) NE6-C-AEMs, and (**h**) NE8-C-AEMs in different voltage ranges. CV curves of NE-C-AEMs voltage ranges of (**i**) 3.8~4.8 V and (**j**) 1.0~4.8 V. (**k**) Rate capabilities of NE-C-AEMs in a current range from 0.2 to 4.0 A·g^−1^ and (**i**) Cycling behaviors over 500 cycles.

The electrochemical performances of the four-series NE-C-AEMs were tested using a 2032 type half-cell configuration with a lithium foil reference/counter electrode in an electrolyte of 1 M LiPF_6_ dissolved in an ethylene carbonate (EC) and dimethyl carbonate (DMC) mixture solvent (1:1 *v*:*v*). Figure 4a–d presents the galvanostatic charge/discharge profiles at a current range from 0.2 to 4 A·g^−1^ over a voltage window between 1.0 and 4.8 V. NE6-C-AEMs showed a high reversible capacity of ~485 mA·h·g^−1^, whereas NE2-C-AEMs exhibited a poor reversible capacity of ~60 mA·h·g^−1^ at a specific current rate of 0.2 A·g^−1^, which is one-eighth that of the NE6-C-AEMs (Figure 4a,c). The one-eighth reversible capacity is difficult to understand considering that the NE2-C-AEMs have a much higher oxygen content (16.2 wt.%) than the NE6-C-AEMs (8.4 wt.%) because the oxygen contents and pseudocapacitive charge storage behaviors are closely related. In addition, in terms of the electrochemical double layer (EDL) capacitance, the large difference in reversible capacities appears to be unconvincing because the specific surface area of NE2-C-AEMs was ~76% that of NE6-C-AEMs. Moreover, the highest specific surface area sample, NE8-C-AEMs, showed a reversible capacity of ~360 mA·h·g^−1^, which is ~74% of that observed for NE6-C-AEMs. These results are inconsistent with the general belief of the correlation between the specific surface area/oxygen content and reversible capacity. To reveal their mutuality, cyclic voltammetry (CV) tests were conducted in several different voltage sections (Figure 4e–h). In the higher voltage section based on the open circuit voltage (OCV, approximately 3.1 V vs Li^+^/Li for NE-C-AEMs), the hexafluorophosphate anion was adsorbed on the surface of the working electrode (NE-C-AEMs) by EDL formation because the anion did not exhibit pseudocapacitive behavior in C-AEMs including oxygen functional groups. Accordingly, a rectangular-like CV curve was obtained in the voltage section of 3.8–4.8 V, wherein their area indicates the specific EDL capacitance. When the voltage range was increased gradually from 1.0 (3.8–4.8 vs Li^+^/Li) to 1.8 V (3.0–4.8 V vs Li^+^/Li), the y-axis scale (specific capacitance) was unchanged and their initial rectangular-like shapes were maintained, indicating that the charge storage mechanism is based on EDL formation. On the other hand, as the voltage range was increased gradually to the voltage section below OCV, the specific capacitance was increasing continuously. The voltage-dependent capacity means the presence of pseudocapacitance, which has a voltage hysteresis. When the voltage range was extended to 3.8 V (1.0–4.8 V vs Li^+^/Li), a maximized CV area was observed owing to the increased pseudocapacitance. Note that in a voltage section of 3.8–4.8 V, the EDL capacitances of NE4-C-AEMs, NE6-C-AEMs, and NE8-C-AEMs were increased gradually with increasing content of activation agent (2 << 4 < 6 < 8) (Figure 4i). This indicates that the capacitive charge storage behaviors in the narrow cathodic voltage range are dependent on the specific surface area of the NE-C-AEMs. In contrast, the overall capacitances over a wide voltage section of 1.0–4.8 V were different from the relationship between the specific surface area and capacitance (Figure 4j). This could be due to the differences in pseudocapacitance. Therefore, both the EASA and redox-active heteroatoms should be considered to affect the overall capacitance. In the case of NE2-C-AEMs, the pore size was too small for the charge carriers to approach most of their internal surfaces, resulting in poor EDL capacitance and pseudocapacitance despite having the highest heteroatom content. In contrast, although NE8-C-AEMs had the lowest heteroatom content, the quantitative pseudocapacitance value was larger than that of NE2-C-AEMs. This could be due to the larger effective heteroatom content on NE8-C-AEMs, which have a more open surface area.

**Figure 5:**
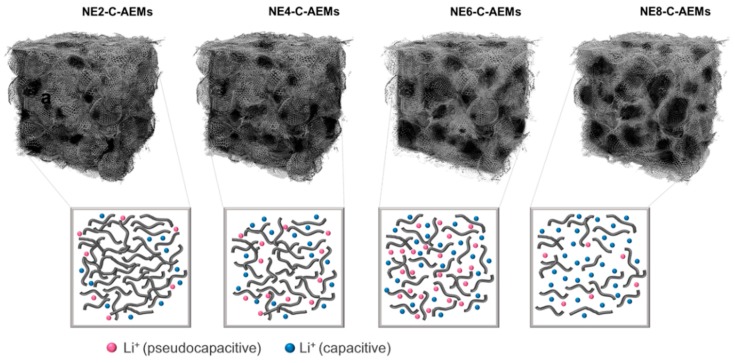
Schematic image showing the relationship between the pore size and charge storage performances on the nanoporous carbon.

The schematic image shown in Figure 5 describes the experimental results. Heteroatoms present in accessible internal surfaces could have redox activity for lithium ions. In contrast, a smaller pore diameter <~2 nm has no redox activity for lithium ions in a positive voltage section as well as EDL capacitance, as observed in the NE2-C-AEMs sample. These results suggest that in a positive voltage section (1.0~4.8 V vs Li^+^/Li), the two factors (≥2 nm nanopores and redox-active heteroatoms) are a core for the pseudocapacitive lithium-ion storage behaviors. Figure 4k presents the rate capabilities characterized in the current range from 0.2 to 4.0 A·g^−1^. The specific capacities of NE-C-AEMs decreased gradually with increasing current density. In addition, the relatively EDL capacitance-dominant NE4-C-AEMs and NE8-C-AEMs showed superior rate capabilities to that of NE6-C-AEMs. At 1.0/4.0 A·g^−1^, 30/15, 201/160, 370/225, and 294/220 mA·h·g^−1^ were achieved for NE2-C-AEMs, NE4-C-AEMs, NE6-C-AEMs, and NE8-C-AEMs, respectively (Figure 4k). The NE6-C-AEMs and NE8-C-AEMs showed higher specific capacities than those (>200 mA·h·g^−1^) of conventional cathode materials, even at high current densities of 4 A·g^−1^, demonstrating the high rate performance of C-AEMs as a positive electrode for LIBs. Moreover, the cycling stabilities of the NE-C-AEMs were maintained for more than 300 cycles (Figure 4l).

**Figure 6:**
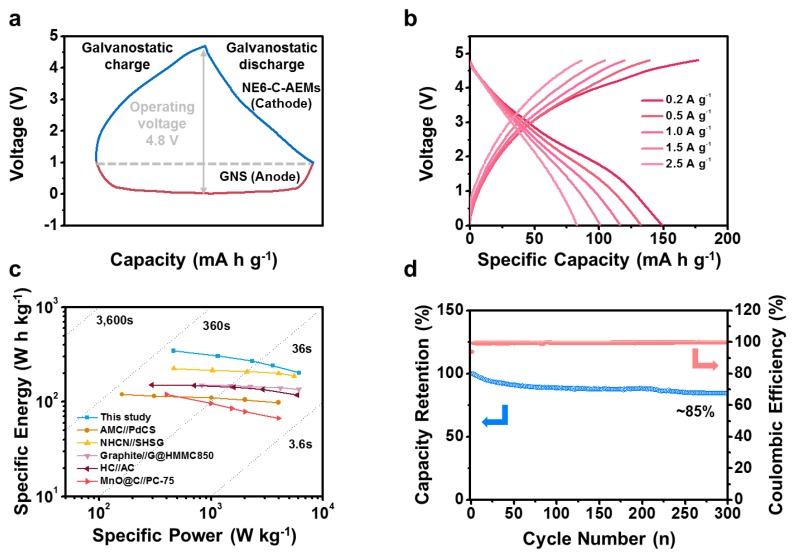
Electrochemical performance of GNS//NE6-C-AEM full cells in an electrolyte of 1 M LiPF_6_ dissolved in EC/DMC mixture solution (1:1 v/v) over a voltage window between 0.01 and 4.8 V. (**a**) Schematic image of the respective galvanostatic voltage profiles of GNS anode and NE6-C-AEM cathode in the full cell system. (**b**) Galvanostatic charge/discharge profiles at different current densities. (**c**) Ragone plots of several energy storage devices, including GNS//NE6-C-AEM cells, and (**d**) capacity retention and coulombic efficiency results over 300 continuous cycles.

**Figure 7:**
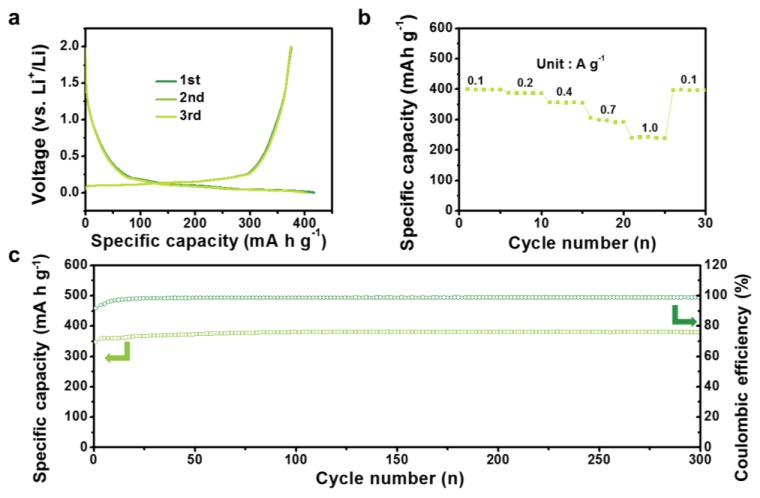
Electrochemical performances of GNS anode in an electrolyte of 1 M LiPF_6_ dissolved in an EC/DMC mixture solution (1:1 v/v) over a voltage window of 0.01~2.0 V vs Li^+^/Li. (**a**) Galvanostatic discharge/charge profiles, (**b**) Rate capabilities in current densities from 0.1 to 1.0 A·g^−1^, and (**c**) Cycling behaviors over 300 cycles.

The feasibility of the high-performance NE6-C-AEMs was demonstrated further by full-cell tests with graphite nanosheet (GNS) anodes, as shown in Figure 6. Before the full-cell test, the NE6-C-AEM cathode and GNS anode were assembled as respective half cells with a lithium metal counter/reference electrode and were precycled for 10 cycles. The precycled electrodes were reassembled as full cells. Figure 7 shows the electrochemical performance of the GNS anode. As shown in Figure 6a, the full cells were operated in a wide voltage window of 0.01~4.8 V at different current ranges. To balance the energy and power capabilities between the GNS anode and NE6-C-AEM cathode, the initial voltage was tuned to 1.0 V, wherein the anode and cathode worked by 0.01 and 4.8 V, respectively. The GNS//NE6-C-AEM cells could deliver specific capacities of ~150 mA·h·g^−1^ at 0.2 A·g^−1^ and ~83 mA·h·g^−1^ at 2.5·A·g^−1^, indicating high energy and power performance (Figure 6b). The energy vs power relationship is depicted as a Ragone plot, as shown in Figure 6c, in which the GNS//NE6-C-AEM cells showed a specific energy of ~345 Wh·kg^−1^ and a specific power of 6100 W·kg^−1^. This is much higher than previously reported results [31,32,33,34,35]. In addition, stable cycling was achieved for more than 300 cycles with 85% capacity retention over 300 cycles (Figure 6d). Although the carbon-based cathode material require a prelithiation process, several methods such as a chemical treatment, electrochemical prelithiation, addition of electrolyte additives, or direct contact to lithium metal demonstrate their feasibility in full-cell systems [36]. Therefore, the carbon-based cathode such as NE6-C-AEMs can be a promising cathode material for LIBs.

## 4. Conclusions

In summary, NE-C-AEMs, which have similar microstructures but different textural and surface properties, were prepared from biowaste-derived precursor materials through controlled activation/carbonation process. Their specific surface area, heteroatom contents, and mean pore radius were 2570~3650 m^2^·g^−1^, C/O ratio of 5.9~28.1, and 3.7 to 6.4 Å, respectively. The electrochemical results of the nanopore-engineered series samples suggest that effective charge storage can occur when the pore size is increased to ≥2 nm. Therefore, a highly activated but heteroatom-rich sample, NE6-C-AEMs, showed the highest specific capacity of ~485 mA·h·g^−1^. The NE6-C-AEMs also showed high rate capability and stable cycling behavior, and the practicality of NE6-C-AEMs was demonstrated through full-cell tests.

## Figures and Tables

**Table 1:** Textural properties of NE-C-AEMS.

Sample	^a^ S_BET_	^b^ V_Tot_	^c^ S_Mic_	^d^ S_Meso_	^e^ V_Mic_	^f^ APS	^g^ APR
NE2-C-AEMs	2566.2	1.09	2320.7	245.5	0.94	23.38	3.65
NE4-C-AEMs	2910	1.4	2317	593	0.98	20.61	4.64
NE6-C-AEMs	3396.2	1.86	2143.3	1252.9	0.91	17.66	5.84
NE8-C-AEMs	3651.5	2.20	2317.5	1334	1.0	16.43	6.40

a: BET surface area (m^2^·g^−1^), b: total pore volume (cm^3^·g^−1^), c: micropore surface area (m^2^·g^−1^), d: mesopore surface area (m^2^·g^−1^), e: micropore volume (cm^3^·g^−1^), f: average particle size (Å), and g: average pore radius (V/A) (Å).

**Table 2:** Chemical structure of NE-C-AEMs characterized by XPS and elemental analysis.

Sample Name	XPS (at.%)			Elemental Analysis (wt.%)		
	C	O	C/O Ratio	C	O	C/O Ratio
NE2-C-AEMs	85.6	14.4	5.9	83.3	16.2	5.1
NE4-C-AEMs	91.4	8.6	10.6	89.0	10.6	8.4
NE6-C-AEMs	95.4	6.4	15.1	91.2	8.4	10.9
NE8-C-AEMs	95.7	3.4	28.1	95.4	4.2	22.7
